# Novel liquid-liquid extraction and self-emulsion methods for simplified isolation of extra-virgin olive oil phenolics with emphasis on (-)-oleocanthal and its oral anti-breast cancer activity

**DOI:** 10.1371/journal.pone.0214798

**Published:** 2019-04-09

**Authors:** Abu Bakar Siddique, Hassan Ebrahim, Mohamed Mohyeldin, Mohammed Qusa, Yazan Batarseh, Ahmed Fayyad, Afsana Tajmim, Sami Nazzal, Amal Kaddoumi, Khalid El Sayed

**Affiliations:** 1 School of Basic Pharmaceutical & Toxicological Sciences, College of Pharmacy, University of Louisiana at Monroe, Monroe, Louisiana, United States of America; 2 Department of Drug Discovery & Development, Harrison School of Pharmacy, Auburn University, Auburn, Alabama, United States of America; VIT University, INDIA

## Abstract

Epidemiological and clinical studies compellingly documented the ability of Mediterranean diet rich in extra-virgin olive oil (EVOO) to reduce breast and colon cancers incidence, cardiovascular diseases, and aging cognitive functions decline. (-)-Oleocanthal (OC) and other EVOO phenolics gain progressive research attention due to their documented biological effects against cancer, inflammations, and Alzheimer’s disease. There is no simple, reliable, and cost-effective isolation protocol for EVOO phenolics, which hinder their therapeutic applications. This study develops novel methods to isolate OC and other EVOO phenolics. This includes the use of ultra-freezing to eliminate most EVOO fats and the successful water capacity to efficiently extract OC and EVOO phenolics as self-emulsified nano-emulsion. Subsequent resin entrapment and size exclusion chromatography afforded individual EVOO phenolics in high purity. OC in vitro and in vivo oral anti-breast cancer (BC) activities validated its lead candidacy. Effective isolation of EVOO phenolics provided in this study will facilitate future preclinical and clinical investigations and stimulate the therapeutic development of these important bioactive natural products.

## Introduction

**Fig 1:**
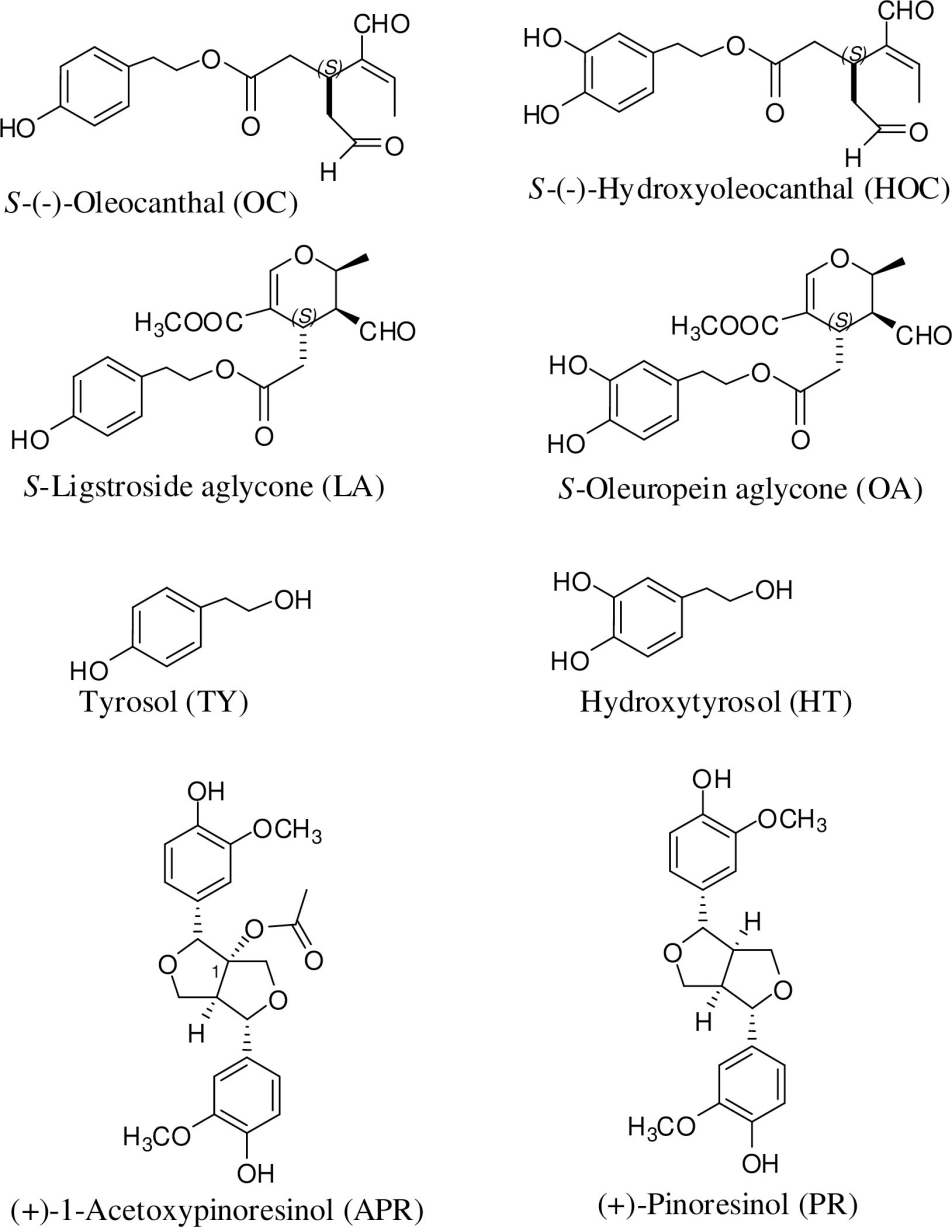
Chemical structures of major EVOO phenolics. *S*-Oleocanthal (OC), *S-*Hydroxyoleocanthal (HOC), *S*-Ligstroside aglycone (LA), *S*-Oleuropein aglycone (OA), Tyrosol (TY), Hydroxytyrosol (HT), (+)-Pinoresinol (PR), (+)-1-Acetoxypinoresinol (APR).

Currently, there is a wealth of epidemiological evidence demonstrating the reduced risk of Mediterranean populations to certain chronic diseases including atherosclerosis, cardiovascular disease, and particular types of cancer, in addition to extended life expectancy compared to other geographical populations [1, 2]. Different statistical and experimental studies revealed a strong correlation between these consistent long-term health-promoting properties and the regular consumption of extra-virgin olive oil (EVOO), which is a major component of the Mediterranean diet [1, 2]. Besides its unsaturated fatty acid content, EVOO contains minor bioactive phenolic ingredients such as: a) simple phenols, including tyrosol and hyroxytyrosol, phenolic acids, and flavonoids, b) lignans, including (+)-pinoresinol (PR) and (+)-acetoxypinoresinol (APR, Fig 1), and c) secoiridoids that are phenolic monoterpenes containing substituted pyran core and represented by *S*-(-)-oleocanthal (OC) (Fig 1), (-)-hydroxyoleocanthal (HOC), *S*-ligstroside aglycone (LA), and *S*-oleuropein aglycone (OA) [1–3].

Among various naturally occurring secoiridoids in EVOO, OC (Fig 1) became renowned for its diverse health-benefiting properties against several ailments [4]. OC was first isolated from EVOO by Beauchamp group with a potent nonsteroidal anti-inflammatory activity due to its cyclooxygenases inhibitory activity [5]. Furthermore, OC has been reported as a potent antioxidant, a neuroprotectant that alters the structure, function, and enhance the clearance of the amyloid-β and reduce Tau hyperphosphorylation associated with the debilitating effects of Alzheimer’s disease [6–9]. OC also inhibited macrophage inflammatory protein-1α (MIP 1α) dysregulated in multiple myeloma [6–9]. The anti-inflammatory activity of OC is mediated, at least in part, through the inhibition of IL-6 expression and secretion and 5-lipoxygenase [6–9]. In addition, the antimicrobial activity of OC against *Helicobacter pylori*, along with its high stability in gastric juice strongly confirmed the OC’s capacity to protect against gastric ulcers and some gastric cancers [10]. On the other hand, OC has been proven to mediate its anticancer effects through the modulation of HGF-c-Met axis and its downstream signaling pathways [11, 12]. Consistently, *in vitro* studies have demonstrated effective inhibition of the proliferation, migration, and invasion of c-Met-dependent human breast and prostate cancer cells, upon OC treatment [11, 12]. In addition, OC markedly reduced tumor volume in triple negative breast cancer (TNBC) mouse model, validating OC lead rank [12]. In support of its health benefits, OC has no toxic effects on the viability and growth of the non-tumorigenic human mammary epithelial cells [13]. Meanwhile, OC caused a marked *in vitro* downregulation of mTOR phosphorylation in TNBC cells, most likely as a downstream effect due to c-Met inhibition [13]. OC inhibited the viability and COX-2 expression in HT-29 colon cancer cells via the activation of AMPK-mediated pathways, plausibly explaining, in part, its preventive and chemotherapeutic potential against colon cancer [14]. OC also inhibited hepatocellular carcinoma (HCC) cells migration and invasion *in vitro* and prevented its lung metastasis *in vivo* [15]. OC inhibited HCC epithelial-mesenchymal transition (EMT) by downregulating Twist, a direct STAT3 target. Recently, OC modulated estrogen receptor expression in luminal breast cancer *in vitro* and *in vivo* and synergized with tamoxifen treatment [16].

Hydroxyoleocanthal (oleacein, HOC, Fig 1) showed antiproliferative, antioxidant, ACE and 5-lipoxygenase inhibitory activities [17–19]. Ligstroside aglycone (LA, Fig 1) induced apoptotic cell death in HER2-dependent breast cancer and showed moderate *in vitro* cytotoxicity against a panel of human cancer cell lines [20]. Oleuropein aglycone (OA, Fig 1) identified as the most *in vitro* potent EVOO phenolic against HER2-dependent breast cancer cell viability by selectively triggering high levels of apoptotic cell death and suppressing lipogenic enzyme fatty acid synthase expression [21, 22].

Lignans including (+)-pinorsinol (PR) and (+)-acetoxypinoresinol (APR, Fig 1) are the second phenolic group in EVOO [23]. PR showed anti-inflammatory activity via inhibition of NO, PGE2, COX-2, TNFα, IL1β, IL-6, and NF-κβ [23, 24]. PR also showed antifungal activity against various *Candida* and *Fusarium* species by inhibiting their trichothecene biosynthesis, antioxidant activity via ROS scavenging and reducing LDL oxidation, hypoglycemic activity via inhibition of α-glucosidase, and *in vitro* neuroprotective effects [23, 24]. Anticancer effects of PR against breast cancer cells proved via reduction of fatty acid synthase (FASN), reducing HER2 expression and activation in HER2-dependent cells [23, 24]. PR inhibited the proliferation of TNBC, prostate, melanoma, ovarian, colon, and leukemia cells *in vitro* [23, 24]. APR exclusively occurs in olive with very limited number of bioactivity studies [23].

Together, this compelling data supported the hypothesis that secoiridoids, specifically OC, and lignans correlate with the positive health outcomes associated with EVOO intake, rendering the discovery of new simplified methods for their isolation appealing. Such reliable isolation method will make EVOO phenolics readily available in large quantities, which will facilitate their future use as pure or combined dietary supplements for improved human health applications.

Currently there are several methods for detection and quantification of EVOO phenolics, including OC, such as direct measurement using q^1^H NMR [3], capillary electrophoresis [25], as well as reversed-phase HPLC methods [26]. Available isolation methods require the use of reversed-phase stationary phases and sophisticated technologies such as HPLC, high performance countercurrent chromatography (HPCCC) [27], as well as several liquid-liquid extraction methods using different organic solvents [11, 28]. In addition, Garcia et al proposed the use of deep eutectic solvents, such as choline chloride in various mixing ratios with sugars, as an alternative to methanol for extraction of EVOO phenolics [29]. On the other hand, Wen et al invented a method for extracting OC from EVOO with high yield using modified starch and performing subcritical liquid extraction with a mixture of organic solvents and spray-drying [30]. The majority of the current methods are focusing on EVOO phenolics/OC detection and quantitation for analytical purposes rather than large-scale isolations. The available methods still have serious drawbacks including the need for sophisticated instruments, massive consumption of organic solvents, high cost, tedious, and time-consuming. Importantly, the majority of EVOO phenolics isolation techniques have poor yields, which hinders research, clinical studies, and commercialization efforts that will require large quantities. OC was previously extracted from EVOO using methanol followed by washing with *n*-hexanes and successive purification of the dried extract using C-18 RP-preparative HPLC [11, 28, 31, 32]. The overall yield was poor due to significant OC loss by C-3, and possibly C-1, acetalization and the process was time-consuming and impractical.

Therefore, the main objective of this study was to develop reliable EVOO phenolics purification techniques useful for practical large-scale use while still time and cost-effective as well as environmentally friendly [33]. Purified OC was emulsified in water and assessed orally against breast cancer in nude mouse xenograft model.

## Materials and methods

### Ethics statement

All animal experiments were approved by the Institutional Animal Care and Use Committee (IACUC), University of Louisiana at Monroe, Protocol number 16MAR-KES-02, and were handled in strict accordance with good animal practice as defined by the NIH guidelines.

### General experimental procedures

TLC analysis was carried out on precoated Si gel 60 F_254_ 500 μm TLC plates (EMD Chemicals), using *n*-hexane-EtOAc (8:2) as a developing system. For column chromatography, Sephadex LH-20 (Sigma Aldrich, bead size 25–100 μ) used for final size exclusion chromatographic purification of EVOO phenolics using isocratic dichloromethane as a mobile phase. 1% Vanillin in concentrated H_2_SO_4_ was used as a visualizing reagent for TLC. ^1^H and ^13^C NMR spectra were recorded in CDCl_3_, using tetramethylsilane (TMS) as an internal standard, on a JEOL Eclipse-ECS NMR spectrometer operating at 400 MHz for ^1^H NMR and 100 MHz for ^13^C NMR. The ESI-MS carried out on AB Sciex-3200 QTRAP LC/MS/MS system (Applied Biosystems, Foster City, CA) using Analyst version 1.4.1 software (MDS Sciex; Toronto, Canada). Analytes were ionized using electrospray ionization (ESI) interfaced with standard turbo V ion source. HPLC analysis conducted using a Shimadzu LC-20AP HPLC system equipped with a variable wavelength UV/Visible detector set at 230 nm. Generally, 1:100 ratio of mixtures to be chromatographed versus the used stationary phase were used in all liquid chromatographic purifications.

### Chemicals

All chemicals purchased from Sigma-Aldrich (St. Louis, MO), unless otherwise stated. EVOO phenolics were isolated from EVOO using the water extraction method similar to that described under sample preparation section. Methanol, acetonitrile, dichloromethane, and ethyl acetate purchased from VWR (Suwanee, GA).

### Extra-virgin olive oil samples

The EVOO samples used in the study were either generously provided by Florida Olive System, Inc., the Dafnis family-Governor, Corfu, Greece, or commercially available and purchased from Brookshire and Sam’s Club chains at Monroe, Louisiana, USA. Samples of eight different varieties including freshly pressed domestic Florida Olive System samples and commercially available imported Brookshire, Batch numbers: L183TE-241, L245TE-241 and Daily Chef, Batch number: LO22RE-565, Tables 1 and 3, and The Governor, Batch number: 5–214000242017 were used in this study. Most of the commercially available EVOO origin was Italy. Olive oil production performed on either two-phase or three-phase mills. All samples provided by small-scale producers that could guarantee their mono-varietal origin.

### Reference compounds

*S*-(-)-Oleocanthal (OC), *S*-(-)-hydroxyoleocanthal (HOC), tyrosol (TY), hydroxytyrosol (HT), *S*-ligstroside aglycone (LA), *S*-oleuropein aglycone (OA), (+)-pinoresinol (PR), and (+)-1-acetoxypinoresinol (APR, Fig 1) were isolated from EVOO (>95% purity, by HPLC analytical conditions) [11, 12]. Separation performed on a Phenomenex Cosmosil 5C_18_-AR-II column (250 x 4.6 mm, 5 μm; Phenomenex Inc., Torrance, CA) at 25 ^0^C. Isocratic elution was performed using H_2_O-CH_3_CN (6:4) as a mobile phase. A flow rate of 1 mL/min, injection volume of 20 μL, and 1 mg/mL sample concentration were used. The identity of each EVOO phenolic was unambiguously defined by extensive 1D and 2D NMR analysis and comparison of its ^1^H and ^13^C NMR data with literature [3]. Pure samples were kept frozen in amber glass vials under N_2_ gas at 0 ^0^C.

### Extraction and sample preparation

#### Acetonitrile ultra-freezing method

**Fig 2:**
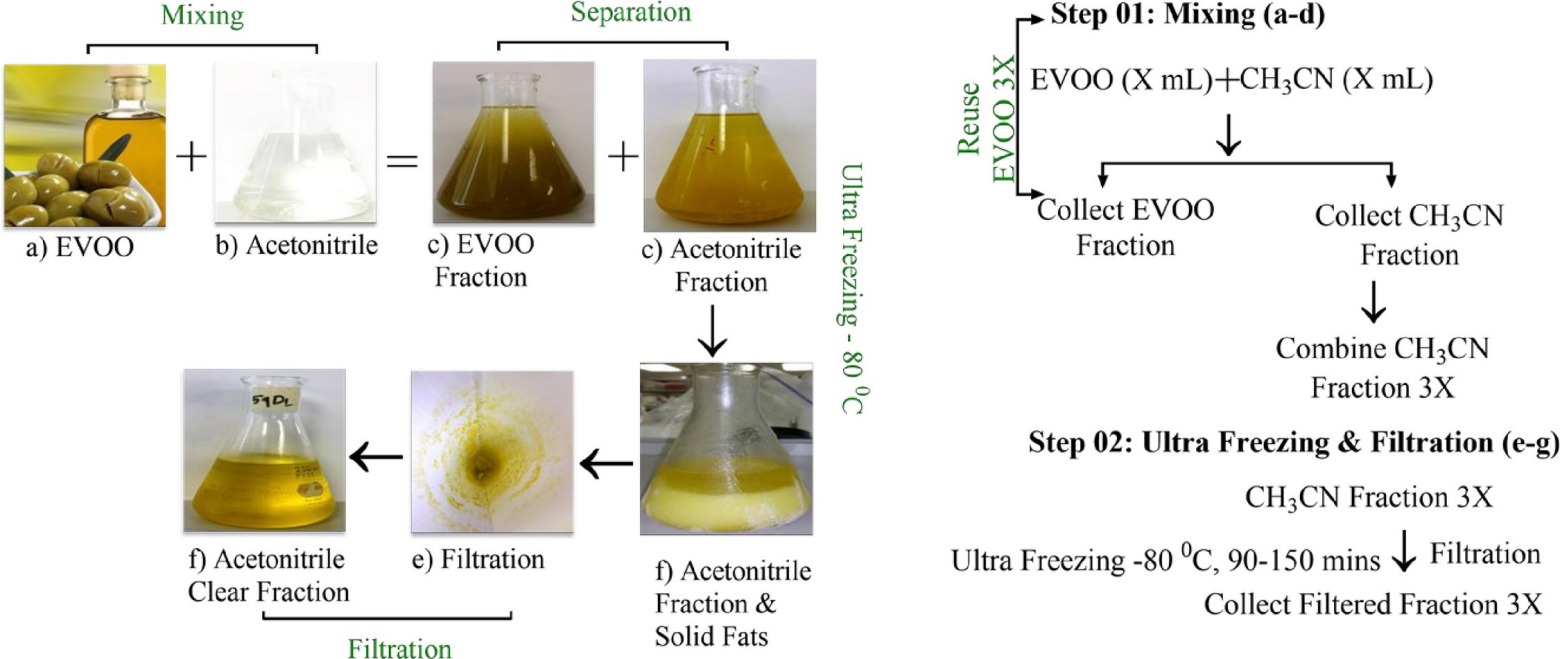
Novel ultra-freezing-based method for EVOO phenolics purification. The sequential steps of mixing, ultra-freezing, and filtration procedures.

About 100 mL EVOO and 100 mL CH_3_CN were mixed in a 500 mL separating funnel, vigorously shaken, and allowed to separate (Fig 2). The process repeated for two additional times; each using 100 mL CH_3_CN. The organic acetonitrile layers were combined and subjected to ultra-freezing for 2 h at -80 ^0^C. The mixture was then immediately filtered on a Whatman #1 filter paper. Ultra-freezing was repeated for two additional times followed by filtration to afford clear oily solution containing OC. This oily solution was then dried and subjected to further purification using lipophilic Sephadex LH20. This method was then repeated to larger EVOO volumes for reproducibility confirmation and large-scale isolations.

#### Water extraction method

**Fig 3:**
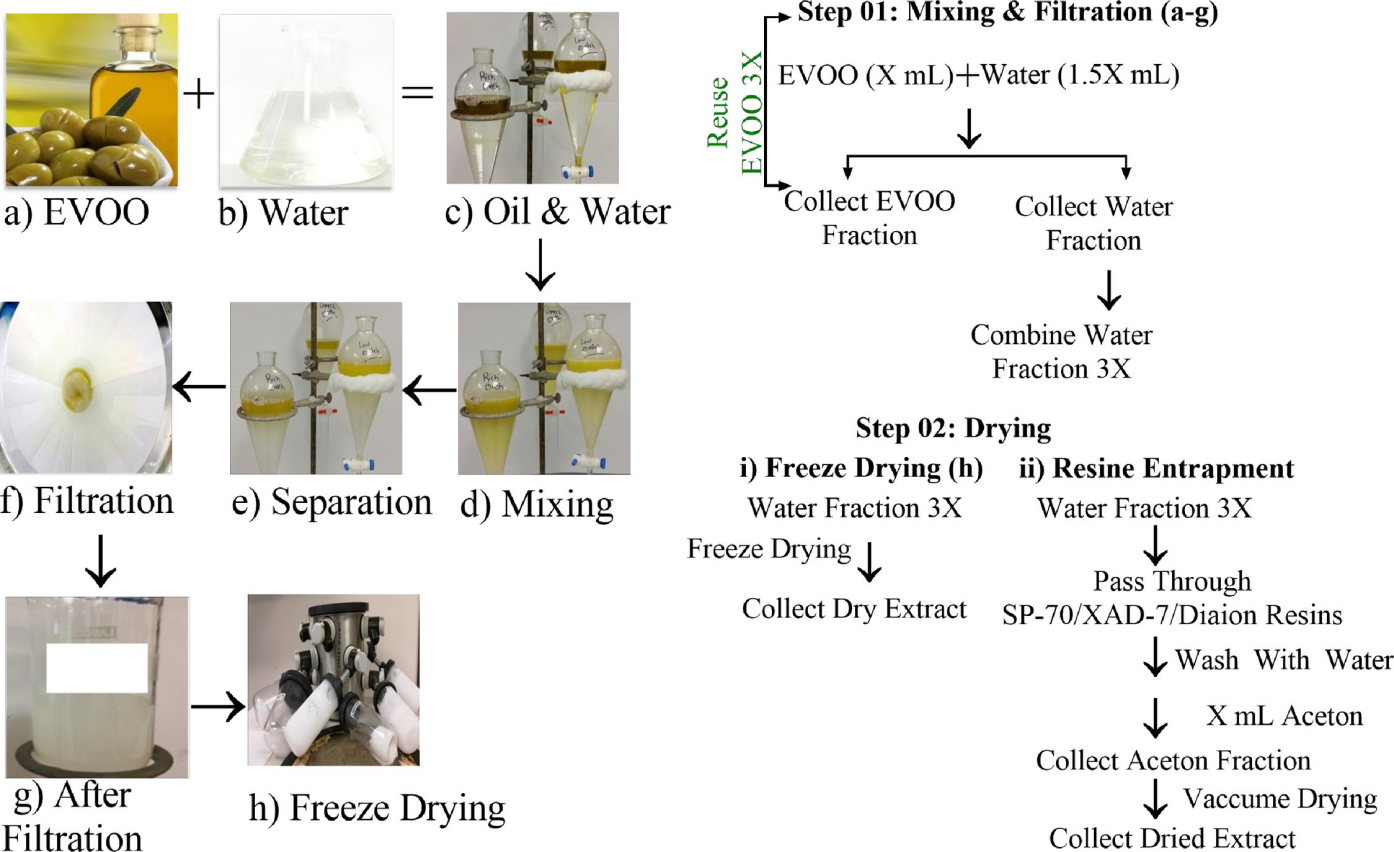
Simple water-based liquid-liquid extraction method for EVOO phenolics isolation. The sequential steps of mixing and filtration procedures.

EVOO (100 mL) mixed with de-ionized water (150 mL) in a 500-mL separating funnel, vigorously shaken three times, and allowed 15–25 min for phase separation. The aqueous extract was then immediately filtered on a Whatman #1 filter paper, followed by freeze-drying until complete dryness (Fig 3). To render the process time and cost-effective, freeze-drying was replaced with a wet-packed column using 45 g Sorbtech (Sorbent Technology Sepabeads Resin Styrenic Adsorbent Sp-70-01, Fig 3). The filtered aqueous extract was then passed through the Sp-70-01 resin column. The column was washed with 150 mL water and finally eluted with 300 mL acetone. The acetone eluate rich in OC and EVOO phenolics was collected and dried under reduced pressure. To confirm the selective EVOO phenolics retention by resin, the eluted water extract was extracted with CH_2_Cl_2_ and subjected to ^1^H NMR analysis, which confirmed the lack of any OC or phenolic contents (S1 Fig). This clearly proven the selective retention of phenolics on resin, which only completely eluted later by acetone. Final purification achieved by subjecting the dried acetone extract to Sephadex LH20 size exclusion chromatography using isocratic CH_2_Cl_2_ as a mobile phase, which eluted PR, APR, LA, and finally OC, respectively. Sephadex LH20 column was then subjected to gradient elution using CH_2_Cl_2_ with increasing amounts of EtOAc (S2 Fig), affording OA, HOC, HT, and TY, respectively. Eluted fractions were monitored using ^1^H NMR (S2 Fig) and ESI mass analysis (S3 Fig). This method is currently a patent pending [33].

### HPLC analysis

**Fig 4:**
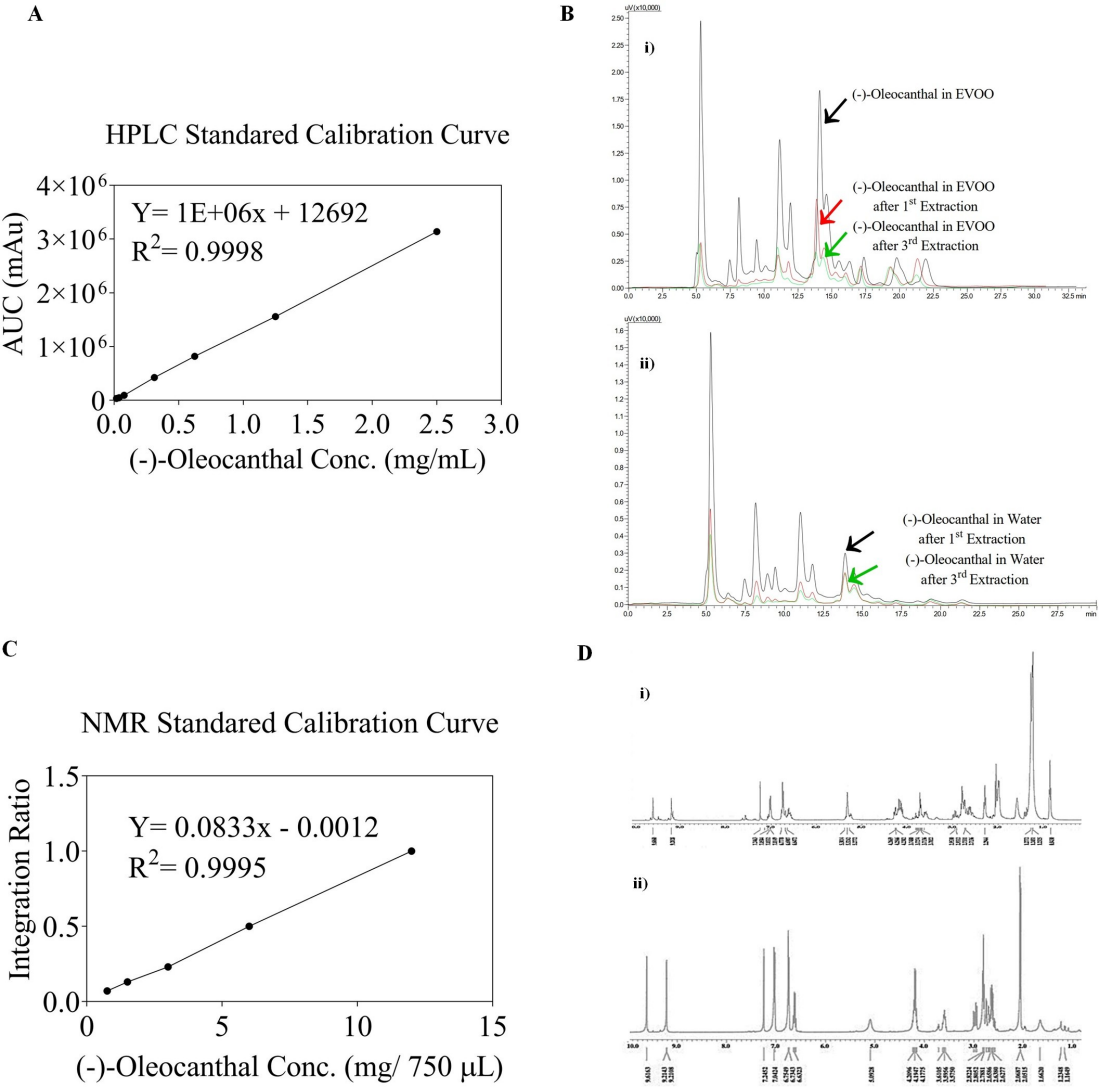
Application of OC-water isolation method on the Governor EVOO batch number: 5–214000242017. A. Oleocanthal standard calibration curve using quantitative HPLC data. B. HPLC Monitoring of water extraction process; i) HPLC chromatogram for crude EVOO and EVOO after water extraction. ii) HPLC chromatogram of the 1^st^ -3^rd^ water extraction. C. OC standard calibration curve using q^1^H NMR data. D. ^1^H NMR-based monitoring of the extraction process. i) ^1^H NMR spectrum of crude water extract showing OC as a major EVOO phenolic ingredient. ii) Pure OC ^1^H NMR spectrum after Sephadex LH20 purification.

EVOO Phenolics identification and quantification were further confirmed using HPLC analysis on a Shimadzu HPLC system equipped with a UV/Visible variable wavelength detector. Briefly, sample extract was dissolved in 100% CH_3_CN. Samples (20 μL) were then injected into Phenomenex Cosmosil 5C_18_-AR-II column (250 x 4.6 mm, 5 μm; Phenomenex Inc., Torrance, CA). The flow rate of the mobile phase (CH_3_CN-H_2_O 1:1, isocratic) was 1.0 mL/min and the analytes simultaneously detected using a UV detector at λ 230 and 254 nm. Elution order was as follows: HT at 8.1 min, APR at 11.1 min, OC at 13.9 min, LA at 19.5 min retention time. Data acquisition and analysis were performed using Lab Solution chromatography software. Quantitatively, calibration curves were prepared using known concentrations of pure OC, as an external standard, and calculating the area under the peak (AUC) for each concentration (Fig 4).

### q^1^H NMR analysis

The OC-rich residue obtained according to previous water extraction procedure, was dissolved in CDCl_3_ (750 μL) and transferred to a 5 mm NMR tube. ^1^H and ^13^C NMR spectra then acquired using tetramethylsilane (TMS) as an internal standard, on a JEOL Eclipse-ECS NMR spectrometer operating at 400 MHz for ^1^H NMR and 100 MHz for ^13^C NMR. Typically, 64 scans were collected into 32K data points over a spectral width of 0−16 ppm with a relaxation delay of 1 s and an acquisition time of 2.1 min for ^1^H NMR. For quantitative ^1^H NMR analysis, calibration curves were established using known concentrations of pure OC as an external standard (Fig 4C). Quantitation was based on the integration ratio of the OC’s key C-3 aldehydic proton signal at 9.23 ppm and residual CHCl_3_ peak in the CDCl_3_ at 7.24 ppm. OC used for the calibration curves was freshly prepared.

### Zeta-potential and particle size analysis of self-emulsified EVOO phenolics/OC extract

The mean particle size of EVOO phenolics self-assembled structures in water was measured by photon correlation spectroscopy (PCS) using a Nicomp TM380 ZLS submicron particle size analyzer (Particle Sizing System, Port Richey, FL) at 25 ^0^C and 90 ^0^C laser light scattering. Samples were diluted with deionized water to avoid multiple scattering and achieve a scattering intensity of 300 kHz. The volume-weighted mean diameter of the particles was calculated based on Stokes–Einstein law by curve fitting of the correlation function. The Zeta-potential of OC/EVOO phenolics self-assembled structures in water was measured using the same instrument under the Zeta mode.

### Short-term stability study

Short-term stability study was conducted by keeping the OC/phenolics emulsion samples at two different temperatures; either RT or 4 ^0^C. After one month, samples were analyzed using HPLC as previously described. In addition, the particle size and Zeta-potential were calculated for the stored samples and compared with those of initial samples prior to storage to assess the stability of OC/phenolics emulsions under different storage and temperature conditions.

### Cell lines and culture conditions

The human BC cell lines MDA-MB-231, MDA-MB-468, BT-474 and MCF-7 were purchased from ATCC (Manassas, VA). All cancer cell lines were maintained in RPMI-1640 (GIBCO-Invitrogen, NY) supplemented with 10% fetal bovine serum (FBS, Gemini Bio-Products), 100 U/mL penicillin G, 100 μg/mL streptomycin and 2 mmol/L glutamine. All cells were maintained at 37 ^0^C in a humidified incubator under 5% CO_2_. The MCF10A cell line, an immortalized, non-tumorigenic human mammary epithelial cell line, was purchased from ATCC. MCF10A cells were maintained in DMEM/F12 supplemented with 5% horse serum, 0.5 μg/mL hydrocortisone, 20 ng/ml EGF, 100 U/mL penicillin, 0.1 mg/ml streptomycin, and 10 μg/mL insulin. All cells were maintained at 37°C in an environment of 95% air and 5% CO_2_ in humidified incubator. Fresh OC water emulsion was prepared by vigorous shaking of Sephadex LH20-purified OC with water to afford a stock solution of 10 mM concentration for all assays. Working solutions at their final concentrations for each assay were prepared in appropriate culture medium immediately prior to use. The vehicle control was prepared by adding the maximum volume of water used to prepare OC treatment concentrations. Pure OC in DMSO also was used as a positive control at 10 μM dose based on earlier studies [11–13, 16, 28].

### MTT proliferation assay

Briefly, MDA-MB-231, MDA-MB-468, BT474, MCF-7 and MCF10A cells, in exponential growth, were seeded at a density of 1×10^4^ cells per well (6 wells/group) in 96-well culture plates and maintained in RPMI-1640 media supplemented with 10% FBS and allowed to adhere overnight at 37 ^0^C under 5% CO_2_ in a humidified incubator [12]. Next day, cells were washed with phosphate buffer saline (PBS), divided into different treatment groups, and then fed defined RPMI-1640 media containing 0.5% FBS, to maintain the viability of the cells throughout the experiment, and experimental treatments, containing designated concentrations of OC or vehicle-treated control media, and incubation resumed at 37 ^0^C under 5% CO_2_ for 48 h. Control and treatment media were then removed, replaced with fresh media, and 50 μL of fresh MTT solution (1 mg/mL) was added to each well and plates were re-incubated for 4 h at 37 ^0^C. The color reaction was stopped by removing the media and adding 100 μL DMSO in each well to dissolve the formed formazan crystals. Incubation at 37 ^0^C was resumed for 20 min to ensure complete dissolution of crystals. Absorbance was determined at λ 570 nm using a plate microreader (BioTek, VT, USA). The % cell survival was calculated as follows: % cell survival = (Cell No. _treatment_/Cell No. _vehicle_) × 100.

### *In vivo* study

#### Animals

Female athymic nude mice (Foxn1^nu^/Foxn^1+^, 4–5 weeks old) purchased from Envigo (Indianapolis, IN). Mice were acclimated to the animal housing facility and maintained under clean room conditions in sterile filter top cages with Alpha-Dri bedding and housed on high efficiency particulate air-filtered ventilated racks at 25 ^0^C, with a relative humidity of 55–65% and a 12 h light/dark cycle, for at least one week before the study. The mice had free access to drinking water and pelleted rodent chow (no. 7012, Harlan/Teklad, Madison, WI).

#### Xenograft model

MDA-MB-231/GFP human breast cancer cells were harvested, pelleted by centrifugation at 850 × g for 5 min, and re-suspended in sterile serum-free DMEM medium (30 μL). Tumor cells’ suspension (1 × 10^6^ cells/50 μL) was inoculated subcutaneously into the second mammary gland fat pad just beneath the nipple of each ketamine-anesthetized animal after anesthesia to generate orthotropic breast tumors [12]. At the beginning mice were randomly divided into two groups: i) the vehicle-treated control group (n = 5), ii) the OC emulsion in water-treated group (n = 5). Treatment (7X/week) started 7 days before-inoculation with orally (p.o.) administered vehicle control (water/saline) or freshly prepared OC water emulsion 10 mg/kg. The mice monitored daily by measuring tumor volume, body weight, and clinical observation. Tumor volume (V) was calculated by V = L/2 × W^2^, where L was the length and W was the width of tumors. The results are presented as average ± SD.

#### Statistical analysis

Differences among various treatment groups were determined by the unpaired t-test using graph pad prism ver. 8. A difference of *p < 0.05, **p < 0.001 was considered statistically significant as compared to the vehicle-treated control group.

## Results and discussion

The isolation of minor bioactive phenolic compounds in EVOO is challenging, time and cost consuming, need significant organic solvent amounts, require sophisticated technologies and reversed phase use, and poor yield due to potential acetalization of secoiridoid aldehydes [3, 27]. Therefore, two simple but effective isolation methods have been developed. It has been well-documented that methanol use for EVOO secoiridoid purification induces immediate and reversible aldehyde acetalization interaction leading to significant yield loss [3]. In order to eliminate the possibility of secoiridoids loss due to the expected acetalization, methanol has been replaced by CH_3_CN in the EVOO liquid-liquid extraction procedure [3]. It is well documented that CH_3_CN does not react with any analyte when used as a solvent for extraction of EVOO [3]. EVOO has been extracted three consecutive times with CH_3_CN for efficient phenolic contents extraction (Fig 2). An ultra-freezing protocol has been then applied to the CH_3_CN fraction via storing at -80 ^0^C for 90–150 min, three consecutive cycles (Fig 2). The major fatty content of the CH_3_CN extract solidified and formed a precipitate under ultra-freezing conditions, while minor phenolics remained soluble in CH_3_CN liquid form. Therefore, enormous fatty acid content amount has been filtered out and eliminated by simple filtration (Fig 2). A clear oily solution containing phenolics was collected after each ultra-freezing cycle (Fig 2) with significant reduction in fatty contents evidenced by ^1^H NMR and HPLC analysis. A final Sephadex LH20 column chromatography purification step described earlier was only needed to timely and cost-effectively obtain pure EVOO phenolics in high purity and yield.

Though the acetonitrile-ultra-freezing method is efficient for extracting EVOO phenolics, it still has some limitation. The capacity of space in the -80 ^0^C facility is limited and not suitable for large-scale isolation. The massive organic solvent use is not environmentally friendly and the exhausted EVOO following CH_3_CN extraction can’t be reused. EVOO phenolics can dissolve in polar as well as non-polar solvents and thus considered amphiphilic. For example, OC specifically underwent a spontaneous interaction with D_2_O forming the more polar acetal/ monohydrate, by water addition to the C-3 aldehyde, which was characterized by ^1^H NMR data [3]. Therefore, EVOO phenolics can reversibly interact with water in a similar manner to form more polar water-soluble acetal forms. Recovery rate of original aldehydic form will be high if the contact with water maintained as short as possible, preferably less than 12 h [3]. Since water is readily available, cost-effective, as well as environment friendly solvent, it has been hypothesized that water can be the appropriate solvent for large-scale extraction of EVOO phenolics. The water-EVOO extraction proposed to selectively dissolves phenolics, leaving crude fatty contents undissolved. Accordingly, EVOO was extracted three consecutive times with water via a simple liquid-liquid extraction protocol and the water fractions were collected (Fig 3). As expected, once EVOO mixed with water, it formed an emulsion thus, the process can be considered as a self-emulsifying extraction method (Fig 3). A quantitative study carried out to determine the amount of OC and other major EVOO phenolics in the resultant emulsion as well as in EVOO before and after water extraction using HPLC (Fig 4, Tables 1 and 2). OC and other phenolics were mostly shifted to the water layer, with negligible amount of fatty acids, dropping out the majority of fats in the oil layer (Fig 4). After three water extraction cycles, only 5–10% of the major EVOO phenolic OC content remained in EVOO compared to the fresh EVOO batch and nearly 90–95% of OC has been switched towards water layer as determined by HPLC (Fig 4). The residual amount of OC left in EVOO can be carried over by additional water extraction cycles. Furthermore, the resultant emulsion was subjected to freeze drying for 24–48 h to identify its ingredients using TLC and q^1^HNMR analysis (Fig 4). The water extracts afforded EVOO phenolics, of which OC was the most major, representing ~80–85% of the extract content, along with ~15% monounsaturated fats and the rest of other minor EVOO phenolics, as suggested by TLC, q-^1^HNMR and confirmed by HPLC analysis (Fig 4). In addition, the percentage recovery of OC using the water self-emulsifying method has been calculated and found to be around 90–100% after the fifth extraction round (Table 1). These results strongly confirm the efficiency of water capacity to extract EVOO phenolics. This novel self-emulsifying extraction method has a significant environmentally friendly advantage over all previously reported methods where organic solvents have been used. The resulting EVOO after water extraction can be reused for food without pungency and bitterness, soap, or cosmetics industry [27, 29, 30].

**Table 1:** Efficiency of water extraction method in diverse EVOO sources ^a^.

Sample #	Olive Oil Brand	Batch #	Source/ Origin	Original OC conc. mg/L^*b*^	Water extraction recovery mg/L^*c*^	Escaped OC un-extracted mg/L	% Recovery	Pure OC quantity mg/L ^*d*^
**1**	FL Olive System (FOS) EVOO	NA	FL, USA	87.28	87.03	1.00	98.86	NP
**2**	FOS EVOO	NA	FL, USA	82.89	80.15	4.00	95.23	NP
**3**	FOS EVOO	NA	FL, USA	14.13	10.81	3.89	73.53	NP
**4**	FOS EVOO	NA	FL, USA	0.00	0.00	NN	NN	NN
**5**	Brookshire EVOO	L183TE-241	Italy	222.61	149.45	73.6	67.13	186.67
**6**	Brookshire EVOO	L245TE-241	Italy	106.82	90.76	16.06	84.96	NP
**7**	Daily Chief EVOO	LO22RE-565	Italy	98.4	96.85	1.55	98.42	83.10
**8**	Corn Oil	NA	TN, USA	0.00	0.00	NN	NN	NN

^*a*^NP: Not proceeded. NA-Not available; NN-Not Needed.

^*b*^Analyzed directly in EVOO sample by HPLC on a Phenomenex Cosmosil 5C_18_-AR-II analytical column and isocratic CH_3_CN-H_2_O 1:1 mobile phase.

^*c*^Quantity considered based on EVOO-Water 1:3 extraction ratio. Higher (>100mg/L) EVOO phenolics concentration will need additional 2–3 extractions to reach 95% recovery.

^*d*^Pure quantity obtained after purification by Sephadex LH-20 column chromatography.

**Table 2:** Analysis of different phenolic ingredients in representative commercial EVOO sources ^a^.

Phenolic Ingredient	EVOO LO22RE-565 mg/L	EVOO L245TE-241 mg/L	EVOO L183TE-241 mg/L
**Hydroxytyrosol**	91.3	95.1	108.3
**Tyrosol (TY)**	75.6	78.8	78.2
**Acetoxypinoresinol (APR)**	83.6	121.2	117.9
**Oleocanthal (OC)**	127.2	106.8	222.6
**Hydroxyoleocanthal (HOC)**	80.9	109.4	61.0
**Ligstroside aglycone (LA)**	82.1	82.4	135.2
**Oleuropein aglycone (OA)**	79.2	125.3	101.8

^*a*^Analysis was conducted using the analytical HPLC method detailed in Materials and Methods.

**Table 3:** Stability of OC in water extracts after one-month storage at room temperature and 4 ^0^C^a^.

Sample #	Oil Brand	Room temp. OC mg/L	% Degradation	4 ^0^C OC mg/L	% Degradation
**1**	EVOO	3.18	96.34	13.34	84.67
**2**	EVOO	3.81	95.23	6.46	91.93
**3**	EVOO	0.00	100	4.07	62.35
**4**	EVOO	NN	NN	NN	NN
**5**	Brookshire EVOO	0.30	99.79	9.04	93.95
**6**	Brookshire EVOO	0.0	100	6.81	92.43
**7**	EVOO	NP	NP	NP	NP
**8**	Corn Oil	NN	NN	NN	NN

^*a*^Starting OC concentration in each sample was those reported in Table 1

NP: Not proceeded, NN-Not Needed.

The self-emulsifying extraction method required multiple freeze-drying hours to evaporate water (Fig 3), which is time-consuming and may slightly affect EVOO phenolics yield. To bypass this drawback and make the water extraction method more efficient, wet packed resin adsorbent beads have been used as freeze-drying alternative to remove water and improve purity (S2 Fig). Three different types of entrapping resins were assessed; SP-70, Amberlite XAD-7, and Diaion HP-20. The EVOO phenolics-containing emulsion passed over a column packed with each resin type in water and the eluent was collected after using water as a mobile phase (S2 Fig). This eluted water was extracted with CH_2_Cl_2_, dried, and subjected to ^1^HNMR analysis, which revealed no phenolics content (S2 Fig). Thus, the three resin types fully entrapped OC and other phenolics when eluted with water (S2 Fig). Interestingly, the water fractions contained only unsaturated fatty glycerides evidenced by their corresponding ^1^HNMR spectra (S2 Fig). The resin-packed columns were finally washed with acetone to elute the entrapped phenolics and the acetone fractions were subjected to ^1^HNMR analysis, which confirmed its phenolics content (S2 Fig, Table 1). Acetone is a non-halogenated, biodegradable, volatile, water miscible, environment friendly solvent, and will not acetalize OC and aldehydic secoiridoids, unlike alcohols. The SP-70 was the most efficient resin to entrap OC and phenolics, followed by Amberlite XAD-7, and finally the Diaion HP-20 resin (S2 Fig). Only a single Sephadex LH20 size exclusion chromatographic column was needed to obtain EVOO phenolics in high purity. The order of elution was lignans: PR, APR, followed by LA, and OC. Elution of hydroxytyrosol-based secoiridoids required the use of increasing amounts of EtOAc in CH_2_Cl_2_ to elute OA, HOC, and finally TY/HT.

In an attempt to validate the new extraction method for OC and phenolics from EVOO it was repeated three times to confirm its reproducibility. The same extraction protocol was used for each of the seven EVOO samples from different sources. For each sample, three water extractions were used. In addition, a corn oil sample has been used as a negative control to evaluate the selectivity of the extraction protocol (Table 1). Quantitative-^1^HNMR as well as HPLC analysis have been used for detection and quantification of the major EVOO phenolics among different tested samples (Fig 4, Table 1). The proposed extraction protocol was efficiently and reproducibly able to extract OC from EVOO samples of different sources including Greece, Italy, and Florida. The Florida Olive Systems EVOO Sample-4 as well as corn oil sample did not demonstrate any appreciable quantities of OC (Table 1). Samples containing <80 mg OC/L EVOO will usually require 3–4 extractions, evidenced by HPLC analysis of each water extract, but OC-rich EVOO samples (>80 mg OC/ L EVOO) will need additional water extractions for complete OC extraction.

**Fig 5:**
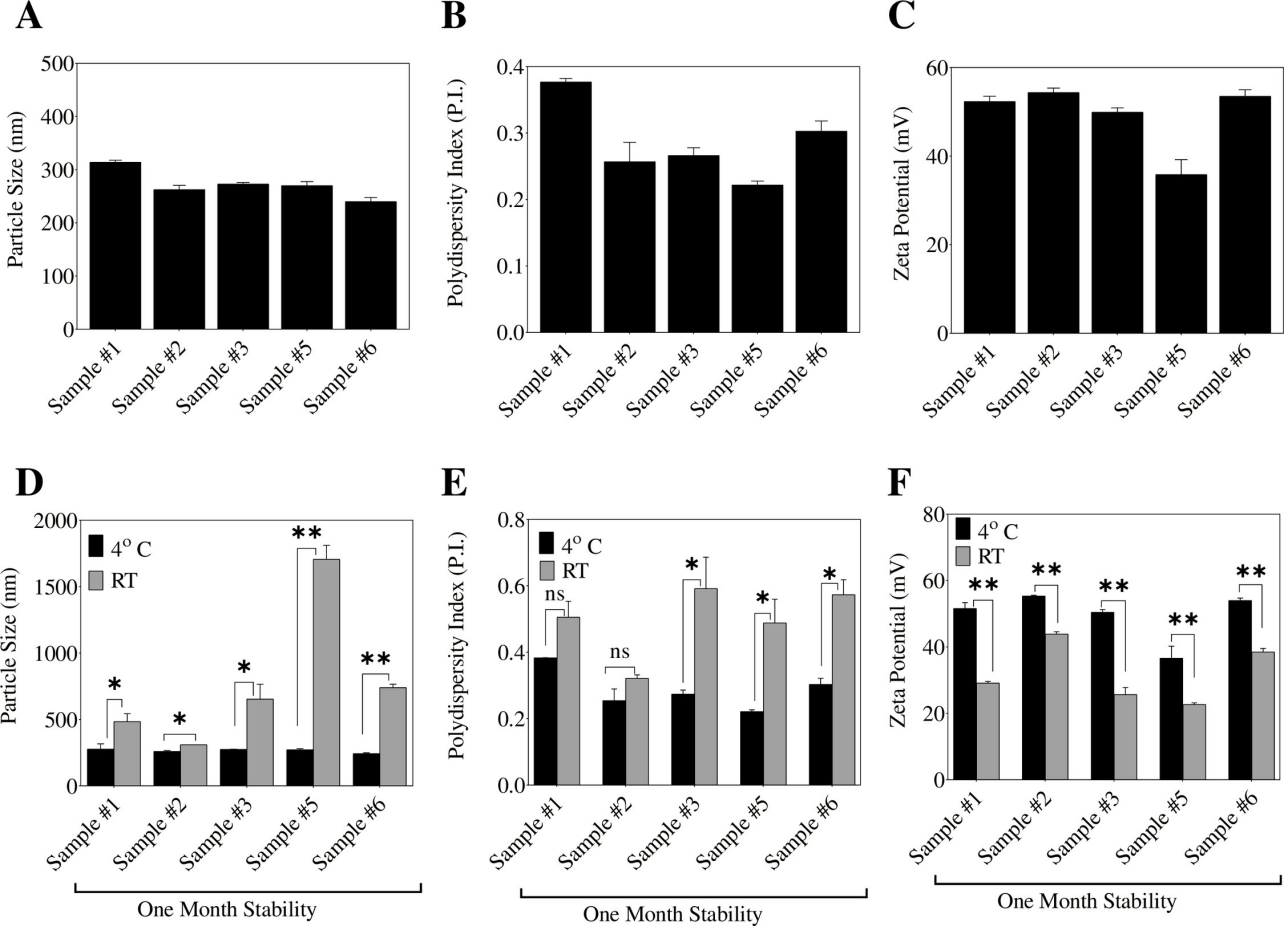
Physical characteristics of OC nano-emulsion using. A. Particle size measurement, B. Polydispersity index, and C. Zeta potential measurement. One-month stability and physical characteristics of water extraction nano-emulsion at RT and 4 ºC using: D. Particle size measurement, E. Polydispersity index, and F. Zeta potential measurement.

Upon water extraction, OC rich water extracts were subjected to further characterization of their emulsion properties including particle size, zeta potential, and polydispersity index (Fig 5). Particle size is a function of volume-weight distribution. Results showed that the water extract of different EVOO samples in solution have excellent nano-system volume-weight distribution of 2.87 nm. The particle size similarity among the water extract of multiple EVOO samples from different sources clearly confirmed the self-emulsifying potential of EVOO phenolics in aqueous solutions.

To confirm the stability of the formed nano-emulsion, the zeta potential of each water extract sample has been determined (Fig 5). Zeta potential serves as a reliable indicator of particle charge and emulsion stability. It reflects the potential difference between the dispersion medium and the stationary layer of fluid attached to the dispersed particle. Nearly all samples had zeta potential >40 mV, suggesting that the self-emulsified phenolics in water is a highly stable nano-emulsion.

In order to evaluate the heterogeneity and correctness of the particle size measurements, the polydispersity index (P.I.) has been determined for each water extract sample (Fig 5). The water extract showed an average P.I. of 0.3, suggesting the uniformity of the measured particle size and implying the lower susceptibility of the nano-emulsion to dispersion (Fig 5). Overall, these results confirm the ability of OC rich water extracts obtained from different EVOO samples to form stable nano-emulsions in aqueous solutions, which might be facilitated by the remained traces of oleic acid triglycerides.

It was interesting to note that different tested samples were able to form stable nano-emulsions with similar particle size, zeta potential and polydispersity index values, suggesting the reproducibility of the OC extraction protocol and eliminating the possibility of any EVOO source-specific results for this method (Fig 5).

The stability of nano-emulsions from different EVOO sources were evaluated by HPLC analysis before and after storing each sample for one month at either RT or 4 ^0^C based on its OC content. In addition, particle size, zeta potential, and polydispersity index have been determined for each nano-emulsion batch after one month to validate the HPLC results (Fig 5). The OC-rich nano-emulsions tend to degrade within one-month storage at RT (Table 3), with significant drop in zeta potential, along with a concomitant increase in particle size and polydispersity index in all investigated EVOO samples (Fig 5). On the contrary, the refrigerated OC nano-emulsion samples at 4 ^0^C showed better stability profile than those stored at RT, with no significant changes in zeta potential, particle size, and polydispersity index, compared to their respective initial values before storage (Fig 5). HPLC analysis showed slow degradation in the refrigerated nano-emulsion samples compared to samples stored at RT (Table 3). Overall, this preliminary stability study suggests that OC/phenolics nano-emulsions are unstable at RT and progressively degrade over time at RT while their stability improved by storage at 4 ^0^C.

**Fig 6:**
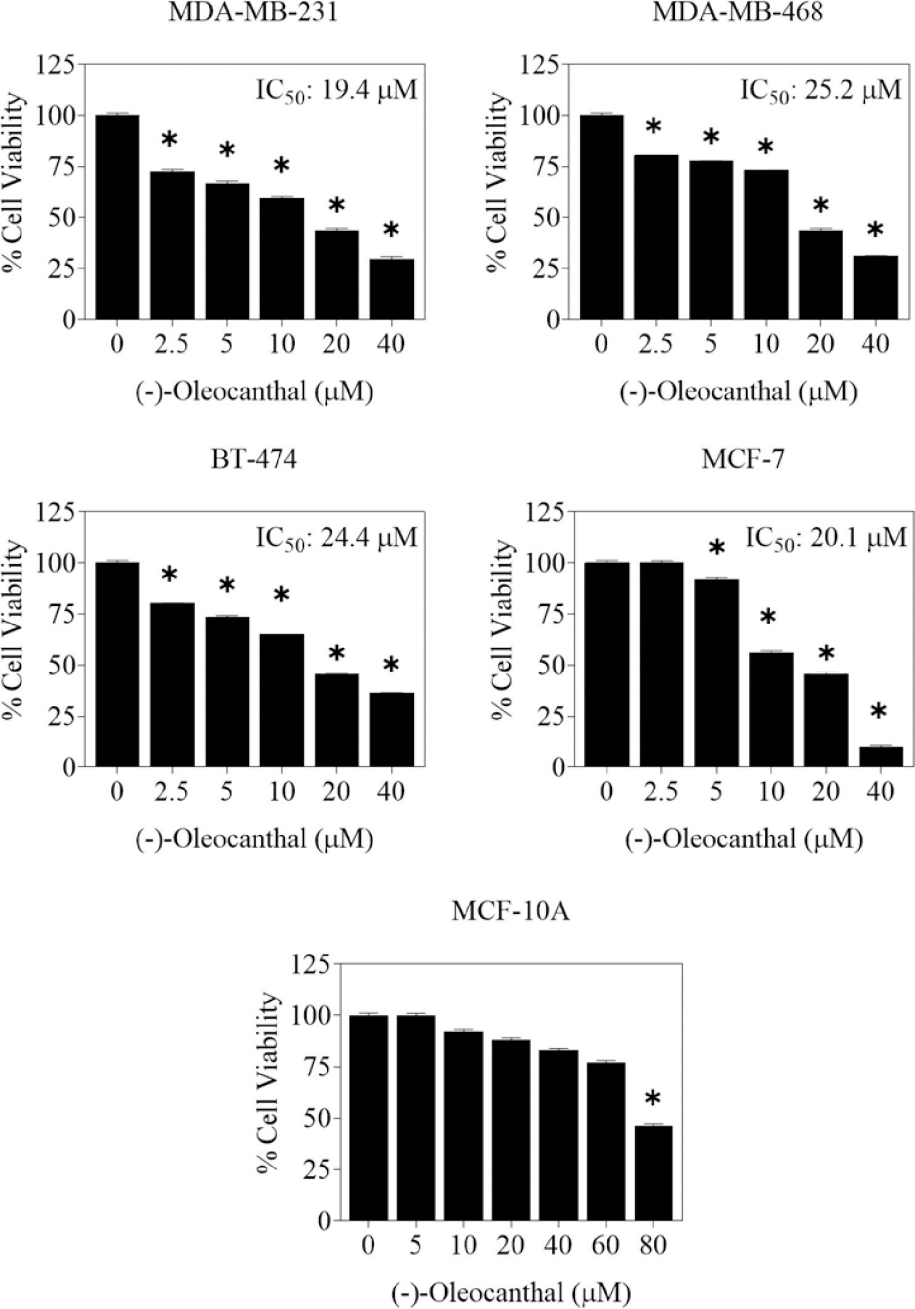
Effect OC nano-emulsion on BC and the non-tumorigenic mammary epithelial cells. BC cell lines MDA-MB-231, MDA-MB-468, BT-474, and MCF-7, in addition to the non-tumorigenic mammary epithelial MCF10A cells were treated by OC nano-emulsion at 72 h treatment period to see the effects of treatment on cells’ growth compared to vehicle control. The non-malignant cells MCF10A was also used to assess treatment selectivity. Viable cell count was determined using MTT assay. Vertical bars indicate the mean cell count ± SD in each treatment group, n = 3/treatment dose. *p <0.05 as compared to vehicle-treated control group.

Sephadex-LH20-purified OC was emulsified in water and assessed for ability to inhibit the proliferation of key human BC cell lines using the MTT assay. The cell lines have been selected to represent different BC phenotypes. The TNBC cells MDA-MB-231 and MDA-MB-468 lack the expression of estrogen receptor (ERα), an important target for hormonal therapy, while they overexpress c-Met, a well-established receptor tyrosine kinase that mediate BC survival and motility and validated as the molecular target of OC in BC [11, 12, 16, 34]. In contrast, MCF-7 and BT-474 BC cells express ERα and c-Met, however, the latter is expressed at relatively lower levels compared to the TNBC cells [12, 16, 34]. The antiproliferative effects of different doses of pure OC emulsion in water on the growth of MDA-MB-231, MDA-MB-468, MCF-7, and BT-474 BC cell lines after 72 h culture period are shown in Fig 6. Treatment with pure OC water emulsion dose-dependently inhibited the proliferation of tested BC cell lines (Fig 6). The IC_50_ values for OC nano-emulsion were 19.4, 25.2, 24.4 and 20.1 μM in MDA-MB-231, MDA-MB-468, BT-474, and MCF-7 cells, respectively (Fig 6). Similarly, effects of OC emulsion on the viability of the human immortalized non-tumorigenic mammary epithelial cells MCF10A was studied to assess its selectivity (Fig 6E). Results showed that treatments with 0–40 μM OC had no effect on MCF10A cell viability compared to vehicle-treated control (Fig 6E). Progressive MCF10A cells growth inhibition was observed only at OC doses higher than 60 μM. These results further suggest the good selectivity of therapeutic OC concentrations toward BC cells at which the non-tumorigenic cells are minimally affected.

**Fig 7:**
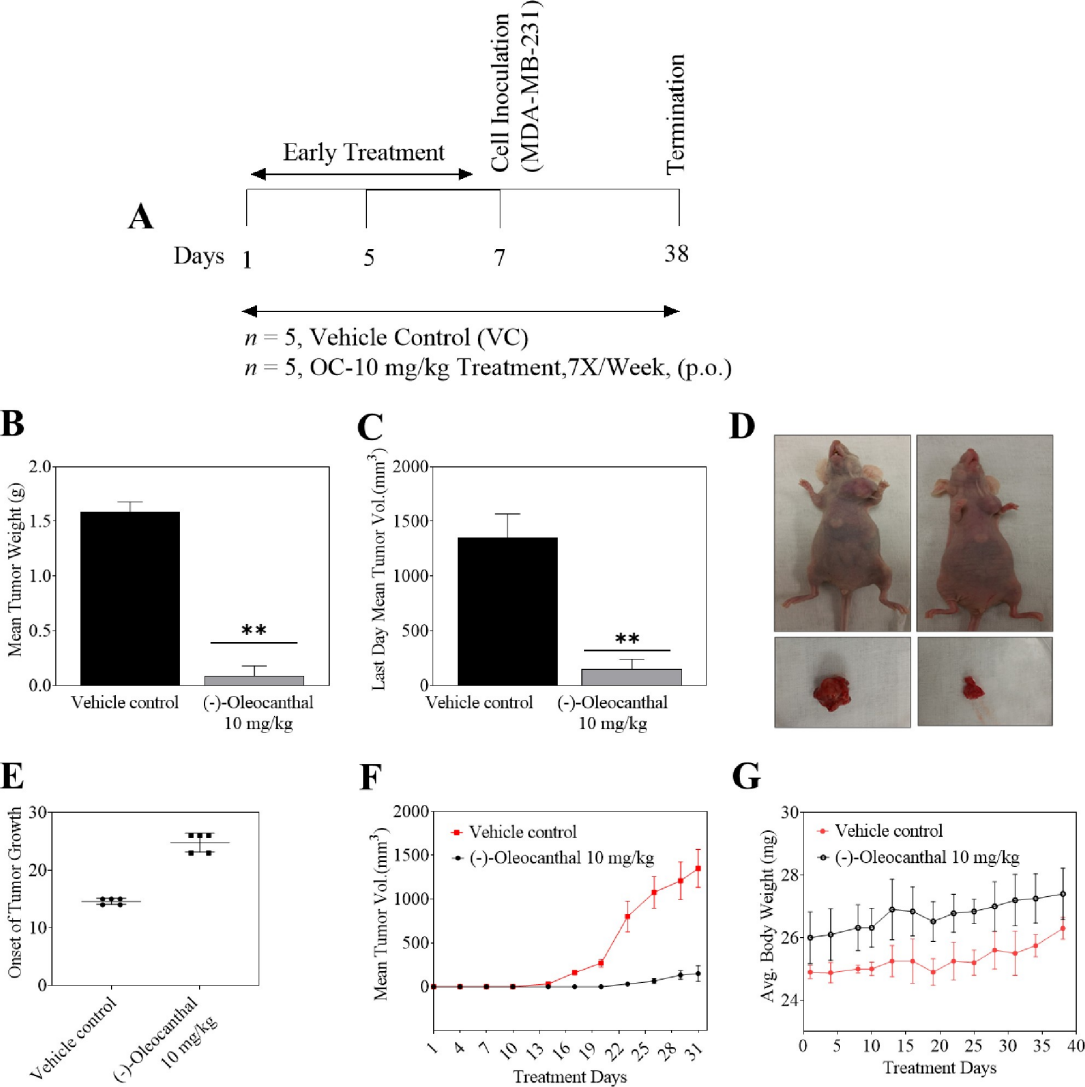
*In vivo* oral activity of pure OC extracted by water extraction method and using water as exclusive vehicle against the human TNBC MDA-MB-231 cells in orthotopic nude mouse xenograft model. A. Design layout of the experiment. B. Vertical bars comparing the mean tumor weight of treated versus vehicle control at the end of the experiment. C. Vertical bars comparing the last day mean tumor volume of treated versus vehicle control at the end of the experiment in each group (n = 5). D. Representative experiment mice and collected tumors at the end of experiment in each group (n = 5). Right mouse: OC-treated orally at 10 mg kg/day, 5X/week. Left mouse: vehicle-treated control. E. Onset of tumor growth. F. Mean tumor volume, n = 5, over the experiment period. Tumor volume (V) calculated using the formula: V = L/2 x W^2^, where L is the length and W is the width of tumors. Collected points represent the mean of tumor volume in mm^3^ in each group (n = 5). G. Monitored mice body weight over the experiment duration. Points represent the mean mice body weight in each group (n = 5) over the experiment duration. Error bars indicate SD for n = 5. **p <0.005 as compared to vehicle-treated control group.

In our previous report, intraperitoneal administration of 5 mg/kg of pure OC, 3X per week showed 65% tumor growth inhibition of the MDA-MB-231 xenograft in female athymic nude mice [12]. OC potency is significantly better when used in early rather than late treatment modes [12]. In the current study oral *in vivo* antitumor efficacy of OC assessed using MDA-MB-231 human BC cells. Freshly prepared OC water emulsion was administered in preventive/early treatment mode orally at 10 mg/kg every day, starting seven days before tumor cell inoculation and continued for 5 weeks. Tumor progression was followed by direct measurement of tumor volume starting 14 days after orthotopic xenografting. Early treatment of OC showed delayed in on set of tumor development in compared to vehicle treated control group (Fig 7). A significant reduction in both tumor volume and tumor weight observed with the OC water emulsion-treated group, compared to the vehicle-treated control group (Fig 7). The 10 mg/kg oral daily OC water emulsion treatments significantly suppressed the MDA-MB-231 tumor growth by 90% on the final day of study, compared to the vehicle-treated control group, without negatively affecting the treated mice’s body weight or their behavior (Fig 7). It is worth noting the significant in vivo activity of OC, unlike its modest in vitro activity, which may suggest possible metabolic bioactivation [12, 15, 16]. This is the first report of oral anti-BC activity of OC. These results indicate that OC emulsion in water demonstrate a robust oral antitumor efficacy in a clinically relevant mouse model, which may also imply good pharmacodynamics effects and tolerability.

## Conclusions

Study results present novel, simplified, environmental friendly, and cost-effective extraction and purification of EVOO phenolics (Figs 3–5), which will motivate future preclinical and clinical investigations of individual olive phenolics and expand their therapeutic applications. The most bioactive EVOO phenolic OC maintained its *in vitro* and oral *in vivo* anticancer activity as freshly prepared nano-emulsion (Figs 6 & 7) suggesting potential future use of this method to develop proper EVOO phenolic formulations for therapeutic and dietary applications.

## Supporting information

S1 Fig ^1^H NMR-guided monitoring of OC entrapment on various resins. ^1^H NMR-guided monitoring of OC entrapment on various resins using JEOL ECS-400 in CDCl_3_. A. ^1^H NMR spectrum of acetone fraction eluted from SP-70. B. ^1^H NMR spectrum of water fraction eluted from SP-70, extracted with CH_2_Cl_2_ and the residue was dissolved in CDCl_3_ and used for analysis. Water-eluted fraction contained only monounsaturated fatty acid glycerides and showed no OC content. C. ^1^H NMR spectrum of acetone fraction eluted from XAD-7. D. ^1^H NMR spectrum of water fraction eluted from XAD-7. Its CH_2_Cl_2_ extract contained only monounsaturated fatty acid glycerides and showed no leftover OC content. E. ^1^H NMR spectrum of acetone fraction eluted from Diaion HP20. F. ^1^H NMR spectrum of water fraction eluted from Diaion HP20, extracted with CH_2_Cl_2_ and the residue contained only monounsaturated fatty acid glycerides and showed no leftover OC content.
(TIF)

S2 Fig ^1^H NMR spectra of various EVOO phenolics. ^1^H NMR spectra of: A) *S*-Oleocanthal (OC), B) *S*-Hydroxyoleocanthal, C) *S*-Ligstroside aglycone, D) *S*-Oleuropein aglycone, E) (+)-Pinoresinol, F) (+)-1-Acetoxypinoresinol.
(TIF)

S3 Fig ESI-Mass spectra of various EVOO phenolics. ESI-Mass spectra of: A) *S*-Oleocanthal, B) *S*-Hydroxyoleocanthal, C) *S*-Ligstroside aglycone, D) *S*-Oleuropein aglycone, E) (+)-Pinoresinol, F) (+)-1-Acetoxypinoresinol.
(TIF)

## Abbreviations

CH_3_CN: Acetonitrile
AMPK: Adenosine Monophosphate-Activated Protein Kinase
BC: Breast Cancer
CO_2_: Carbon dioxide
COX-2: Cyclooxygenase-2
DNA: Deoxyribonucleic Acid
D_2_O: Deuterated Water
CDCl_3_: Deuterated Chloroform
CH_2_Cl_2_: Dichloromethane
DMEM: Dulbecco's Modified Eagle's Medium
EMT: Epithelial-Mesenchymal Transition
ERα: Estrogen Receptor Alpha
FBS: Fetal Bovine Serum
HCC: Hepatocellular Carcinoma
EVOO: Extra Virgin Olive Oil
HPLC: High Performance liquid Chromatography
HPCCC: High Performance Countercurrent Chromatography
MIP: Macrophage Inflammatory Protein
IL-6: Interleukin 6
MMP2: Matrix Metalloproteinase 2
mTOR: Mammalian Target of Rapamycin
Met: Mesenchymal to Epithelial Transition
MTT: 3-(4,5-Dimethylthiazol-2-Yl)-2,5-diphenyltetrazolium bromide
NIH: National Institutes of Health
NMR: Nuclear Magnetic Resonance
OC: Oleocanthal
PBS: Phosphate Buffer Saline
PCS: Photon Correlation Spectroscopy
P.I.: Polydispersity Index
q^1^H NMR: Quantitative proton nuclear magnetic resonance
RPMI 1640, rt: Room temperature
Roswell: Park Memorial Institute
STAT3: Signal Transducer and Activator of Transcription 3
TLC: Thin Layer Chromatography
TMS: Tetramethylsilane
