# Supplementary figures and images for: Novel liquid-liquid extraction and self-emulsion methods for simplified isolation of extra-virgin olive oil phenolics with emphasis on (-)-oleocanthal and its oral anti-breast cancer activity
Source: PLoS One. 2019 Apr 9;14(4):e0214798. doi: 10.1371/journal.pone.0214798 (PMC6456230; doi:10.1371/journal.pone.0214798)

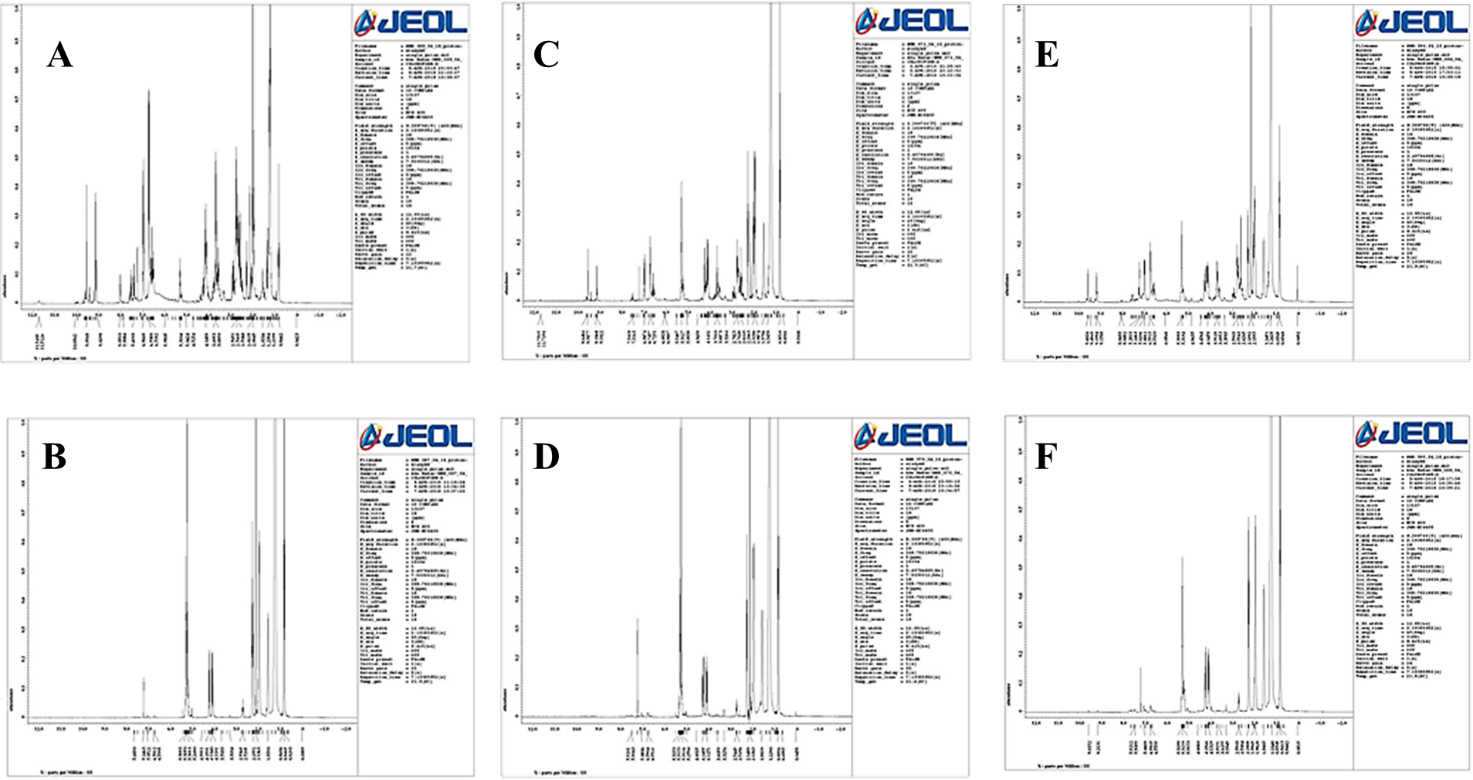

Supplement: S1 Fig — 1H NMR-guided monitoring of OC entrapment on various resins using JEOL ECS-400 in CDCl3. A. 1H NMR spectrum of acetone fraction eluted from SP-70. B. 1H NMR spectrum of water fraction eluted from SP-70, extracted with CH2Cl2 and the residue was dissolved in CDCl3 and used for analysis. Water-eluted fraction contained only monounsaturated fatty acid glycerides and showed no OC content. C. 1H NMR spectrum of acetone fraction eluted from XAD-7. D. 1H NMR spectrum of water fraction eluted from XAD-7. Its CH2Cl2 extract contained only monounsaturated fatty acid glycerides and showed no leftover OC content. E. 1H NMR spectrum of acetone fraction eluted from Diaion HP20. F. 1H NMR spectrum of water fraction eluted from Diaion HP20, extracted with CH2Cl2 and the residue contained only monounsaturated fatty acid glycerides and showed no leftover OC content. (TIF) [file pone.0214798.s001.tif]

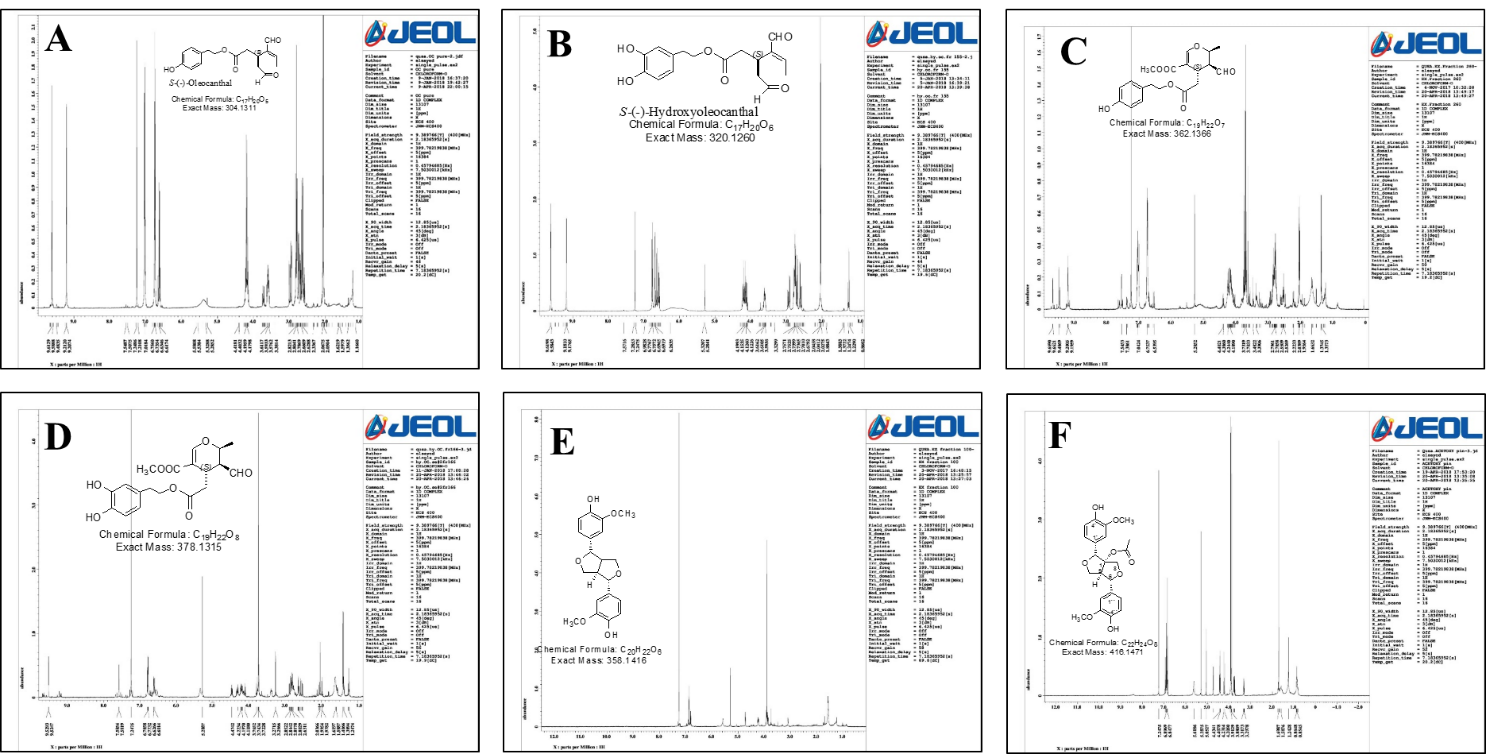

Supplement: S2 Fig — 1H NMR spectra of: A) S-Oleocanthal (OC), B) S-Hydroxyoleocanthal, C) S-Ligstroside aglycone, D) S-Oleuropein aglycone, E) (+)-Pinoresinol, F) (+)-1-Acetoxypinoresinol. (TIF) [file pone.0214798.s002.tif]

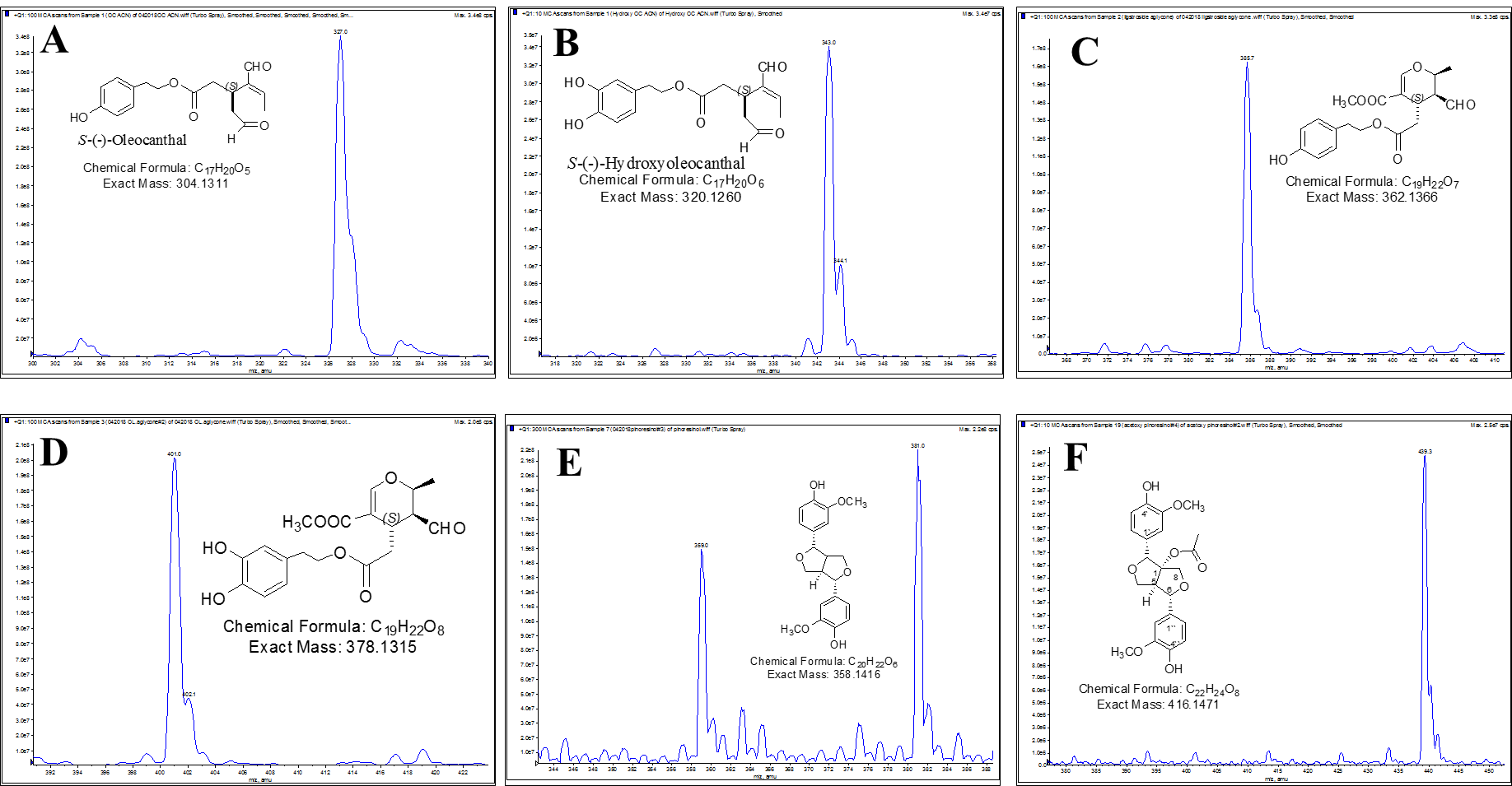

Supplement: S3 Fig — ESI-Mass spectra of: A) S-Oleocanthal, B) S-Hydroxyoleocanthal, C) S-Ligstroside aglycone, D) S-Oleuropein aglycone, E) (+)-Pinoresinol, F) (+)-1-Acetoxypinoresinol. (TIF) [file pone.0214798.s003.tif]
